# Endometritis - Diagnosis,Treatment and its impact on fertility - A Scoping Review

**DOI:** 10.5935/1518-0557.20220015

**Published:** 2022

**Authors:** Neeta Singh, Ankita Sethi

**Affiliations:** 1 Department of Obstetrics and Gynaecology, All India Institute of Medical Sciences, New Delhi, India

**Keywords:** acute endometritis, chronic endometritis, infertility, Asherman’s syndrome, thin endometrium

## Abstract

Endometritis is defined as an infection or inflammation of the endometrium. Endometritis is of two types: acute and chronic. Acute endometritis is the symptomatic acute inflammation of the endometrium, which upon examination with a microscope shows micro-abscess and neutrophil invasion in the superficial endometrium. One of its most common manifestations is postpartum endometritis. Chronic endometritis is a silent disease usually diagnosed on the workup of secondary amenorrhoea and infertility. An important cause of chronic endometritis is tuberculosis, especially in developing nations. Chronic and acute endometritis have been associated with poor reproductive outcomes. Worse outcomes have been reported for individuals with chronic endometritis. This is a scoping review of endometritis and its impact on fertility.

## INTRODUCTION

Endometritis is defined as an infection or inflammation of the endometrium. The normal endometrium does not harbour any microorganisms, but microbes from the cervix and vagina can ascend upwards and lead to inflammation and infection of the endometrium. A very common cause of postpartum endometritis is preterm prelabour rupture of membranes (PPROM) (Sherman *et al*., 1999). Puerperal/Postpartum endometritis is more frequently seen in patients with caesarean sections than normal vaginal delivery and is polymicrobial in nature (Chaim *et al*., 2000). Chronic endometritis is a silent disease usually diagnosed on the workup of secondary amenorrhoea and infertility.

## METHODS

A literature search was performed on the following databases: MEDLINE, Google Scholar, Scopus, EMBASE, Global health, the COCHRANE library, and Web of Science. We searched these databases for studies published until July 2020 in the English language. The literature search was conducted using the combination of the following Medical Subject headings (MeSH) and relevant keywords in different orders: “endometritis”, “acute”, “chronic”, “management”, “diagnosis”, “immunohistochemical”, “hysteroscopy”, “medical management”, “tubercular”, “Asherman’s”, “infertility”, “pathophysiology” and “reproductive outcome”. The reference lists of the included studies were also checked to look for studies that were not found in the electronic literature search. A total of 328 articles were found pertaining to endometritis. Original articles and some review articles published within the last five years were given priority. All articles were accessed in full text. In this review, individual data sources were not sought for, and a descriptive analysis was done. The data were summarized in the form of a descriptive review.

## DISCUSSION

Endometritis can be divided into two subcategories based on histopathology, as acute or chronic endometritis.

### 1 Acute Endometritis

#### 1.1 Definition and Clinical presentation

Acute Endometritis is characterized in histopathology by micro-abscesses in the endometrium and presence of neutrophils in the superficial epithelium and in the lumen of the glands of the endometrium. Acute endometritis presents with symptoms such as fever, pelvic pain, increased vaginal discharge, bad odour, unusual consistency and colour, abdominal pain and distension, abnormal vaginal bleeding, abnormal bowel movements, and generalised malaise.

Group A streptococcus endometritis presents with pain, diarrhoea and vaginal discharge, and may progress to sepsis, toxic shock and necrotising fasciitis. Therefore, these patients should be treated with utmost care. On clinical examination, patients may present with vaginal discharge, uterine or cervical tenderness, and decreased bowel sounds in case of pelvic abscess. According to the CDC (Centers for Disease Control and Prevention) guidelines, a diagnosis of acute PID (Pelvic inflammatory disease) requires the identification of at least one of the following clinical findings: adnexal or cervical or uterine tenderness.

#### 1.2 Etiopathogenesis

Endometritis results from the ascension of bacteria from the cervix and vagina into the uterus. The uterus does not harbour microorganisms until the amniotic sac ruptures, which thus provides passage for bacteria to ascend into the uterus. Microorganisms tend to harbour in an endometrium that is then devitalized and injured (such as in case of a caesarean section or uterine surgery). In any pelvic procedure, if proper asepsis is not maintained or if the woman has an untreated vaginal infection prior to a pelvic intervention such as dilatation, curettage, or endometrial aspiration, then the risk of endometritis is higher. Patients without risk factors may still have endometritis following normal spontaneous vaginal delivery, with an incidence of 1-2% (Boggess *et al*., 2017). Patients with risk factors incidence increase to 5-6%. Risk factors include being a young female from a lower socioeconomic status, having a high BMI (body mass index), prolonged rupture of membranes, repeated per-vaginal examinations, fetal scalp sampling/monitoring, chorioamnionitis, meconium-stained amniotic fluid, and undiagnosed untreated vaginal infection (Boggess *et al*., 2017). The route of delivery is the most important risk factor. Caesarean deliveries have higher risk of endometritis than normal vaginal delivery (Karsnitz, 2013).

**Acute Endometritis:** Acute infections can be caused by both aerobes and anaerobes. Post-caesarean section endometritis is generally due to *Streptococcus pyogenes* and *Staphylococcus aureus infection. Chlamydia* endometritis has a late presentation and generally manifests seven days after delivery (Morgan & Roberts, 2013). Table 1 enumerates the aerobic and anaerobic bacteria that may cause acute endometritis.

**Table 1:** Aerobic and anaerobic bacteria causing acute endometritis

Aerobic Bacteria	Anaerobic Bacteria
• group A S*treptococci* • group B S*treptococci* • *Staphylococcus* • *E.coli* • *Klebsiella pneumoniae* • *Enterococcus* • *Proteus*	• *Peptostreptococcus* • *Peptococcus* • *Bacteroides* • *Prevotella* • *Clostridium*

#### 1.3 Laboratory Evaluation

Diagnostic investigations for acute endometritis include total leucocyte count, swab culture from cervix, and microscopic examination of vaginal discharge samples. Patients unresponsive to treatment may have a pelvic abscess; these cases require a laparoscopic procedure to drain the abscess, followed by intravenous antibiotics in the postoperative period. Proper clinical evaluation and diagnosis are required, since wrong diagnosis and treatment of PID (Pelvic inflammatory disease) unnecessarily hampers patient quality of life. For instance, signs of cervicitis or vaginitis along with one of the minimum criteria, increases the probability of an accurate diagnosis. Table 2 enumerates the findings and the clinical criteria that increase the specificity of a diagnosis of PID as per the CDC guidelines (2015) (Workowski & Bolan, 2015). Table 3 enumerates the most specific findings for the diagnosis of PID.

**Table 2:** As per the CDC (Centers for Disease Control and Prevention) guidelines (2015) (Workowski & Bolan, 2015), one of the findings listed below along with clinical criteria increase the specificity of diagnosing Pelvic Inflammatory Disease

Pelvic Inflammatory Disease: findings
• *Neisseria Gonorrhoeae* or *Chlamydia Trachomatis cervical infection*
• Elevated ESR (Erythrocyte Sedimentation rate)
• Elevated CRP (C-Reactive Protein)
• Abnormal cervical discharge
• Abundant WBCs (White blood cells) in vaginal fluid on saline microscopy
• Fever (temperature >101°F />38.3°C)

**Table 3:** The most specific findings for the diagnosis of PID (Pelvic Inflammatory Disease)

The following findings are the most specific for arriving at a diagnosis of PID:
• Histopathology diagnosis of endometritis on endometrial aspirate tissue sample.
• Hydrosalpinx with or without free pelvic fluid on transvaginal sonogram or
• MRI showing TO (tubo-ovarian) mass, or
• Doppler studies suggestive of pelvic infection (e.g. tubal hyperaemia)
• Hysterosalpingography (HSG) is not recommended in acute infection, but if HSG is done then irregularity of the contour of the endometrial cavity and intravasation of contrast into the vascular and lymphatic system is sign of acute endometritis. • Acute Salpingitis is identified by a ragged contour of the lumen of the tubes and diverticular outpouchings on HSG. Pelvic tuberculosis leads to oedematous thickening of the walls of the tubes and dilatation. The tubes are dilated, convoluted and form a C or S shape. On HSG, tubercular salpingitis presents as hydrosalpinx, beaded tubes (lead pipe appearance).
• Hysteroscopy is not recommended in acute infection (endometritis/salpingitis) • In chronic endometritis, hysteroscopic features: endometrial micropolyposis, they are multiple 1-2 mm sized protrusions or polyps arising from the endometrium with associated endometrial stromal thickening and oedema
• Laparoscopically proven signs of PID

In women undergoing a diagnostic laparoscopy for PID, endometrial aspirate should be taken if there is no sign of salpingitis, since in some women endometritis alone may be present without signs of PID.

#### 1.4 Imaging

Ultrasound helps in the diagnosis of postpartum patients with abdominal pain and fever. Ultrasound findings of endometritis are a thickened, heterogeneous endometrium, fluid collection in the uterus, and foci of air in the uterus (Karsnitz, 2013).

Differential diagnosis includes retained products of conception, infected blood collection (as blood is a good culture media for any bacteria), and pus collection.

Clots and debris may be present in up to 24% of postpartum patients (Plunk *et al*., 2013). In normal and healthy postpartum patients, ultrasound examination may find gas in the endometrium up to three weeks after delivery. The ultrasound findings used to diagnose endometritis may also be seen in normal postpartum patients; therefore, good clinical acumen is required to arrive at the final diagnosis. A patient with endometritis may have a normal pelvic ultrasound. CT scans can show infection and inflammation of the surrounding uterine tissue or parametrium (Nalaboff *et al*., 2001; Vandermeermd & Wong-You-Cheong, 2010; Plunk *et al*., 2013; Laifer-Narin *et al*., 2014).

#### 1.5 Treatment

Apart from symptomatic management, rest, adequate hydration and antibiotics need to be started immediately through the intravenous route for the first 48 hours, followed by oral antibiotics in cases of severe infection; otherwise, oral antibiotics should be given to patients with mild to moderate disease. Simultaneously, sexual partner/s need to be treated and advised on use of barrier contraceptives (Workowski & Bolan, 2015).

Treatment of acute endometritis should provide broad spectrum coverage against the pathogens most likely causing the infection. Treatment should begin as early as possible, as this helps to prevent of long-term complications. Appropriate treatment regimen selection depend on availability, acceptability, and cost. In mild and moderate PID, parenteral and oral treatment regimens are equally efficacious. The need of hospitalisation should be decided based on clinical assessment (Workowski & Bolan, 2015). Table 4 enumerates the factors that, when associated with acute endometritis, decide in favour of patient hospitalisation.

**Table 4:** Factors that decide for need of hospitalization

Hospitalization
Acute abdomen (Surgical emergency)
TO or pelvic abscess
PID in Pregnancy
High grade fever
Excessive nausea and vomiting
Non-compliance with oral regimen
No improvement to oral antibiotics

*Parenteral Treatment* (Workowski & Bolan, 2015)

Oral Medications can be started usually within 24 to 48 hours of observable clinical improvement. Presence of tubo-ovarian abscesses mandates hospital admission and observation for at least 24 hours.

Recommended Parenteral Regimens according to CDC 2015 guidelines are:

- Inj Cefotetan 2g iv BD (twice a day) or Inj Cefoxitin 2 g iv QID (four times a day) plus Tab Doxycycline 100 mg BD
- Tab doxycycline 100 mg BD is given orally after 24-48 hours of clinical improvement to complete the two weeks therapy or
- Inj Clindamycin 900mg intravenous (iv) every 8 hours along with inj Gentamycin loading dose iv (2 mg/kg), (followed by a maintenance dose of 1.5 mg/kg) every 8 hours. Single daily dosing (3-5 mg/kg) can also be substituted alternatively.
- Oral therapy with Tab Clindamycin 450 mg QID PO (per orally) or Tab Doxycycline 100 mg BD can be used to complete the two weeks therapy

*Intramuscular* (IM) and *Oral Treatment* (Workowski & Bolan, 2015)

Oral or Intramuscular treatment are recommended and can be administered to patients with mild or moderate acute PID. Intramuscular or oral therapy produces the same clinical outcomes in patients with mild or moderate PID. Patients not responding to oral/IM treatment within 72 hours should be reassessed and given intravenous therapy.

Recommended Regimens

- Inj Ceftriaxone 250mg i.m. single dose and Tab Doxycycline 100 mg BD with or without Tab Metronidazole 500 mg BD for 2 weeks
- Single dose of Inj Cefoxitin 2 g i.m. and Probenecid 1 g orally and Tab Doxycycline 100 mg BD for 2 weeks with or without Tab Metronidazole 500 mg BD for 2 weeks
- Injectable third generation cephalosporin and Tab Doxycycline 100 mg BD for 2 weeks with or without Tab Metronidazole 500 mg BD for 2 weeks

#### 1.6 Complications

One to four per cent of patients with acute PID may have complications such as pelvic peritonitis, pelvic abscess, septicaemia, septic shock, septic pelvic thrombophlebitis and necrotizing fasciitis, which may lead to uterine necrosis and eventually hysterectomy for the infection to resolve. Pus drainage or a surgical intervention such as pigtail insertion may be required if the infection has produced a drainable fluid collection (Karsnitz, 2013). In the long run, if not treated properly or incompletely, patients may develop pelvic adhesions, distortion of the pelvic anatomy, disturbed tubo-ovarian relationship, and intrauterine adhesions, which may eventually lead to infertility. Acute endometritis may lead to Asherman’s syndrome and eventually uterine factor infertility or secondary amenorrhoea.

#### 1.7 Prevention

Endometritis can be prevented by early detection and management of STIs (Sexually transmitted infections), safer sex practices, sterile techniques during pelvic procedures such as vaginal delivery, C-section, abortions, etc.

#### 1.8 Differential Diagnosis

Whenever a postpartum patient presents with postpartum fever and abdominal pain, the following conditions should be considered in differential diagnosis: lower urinary tract infections, pneumonia, and septic pelvic thrombophlebitis. If the patient does not improve clinically even after antibiotic and/or surgical management for endometritis, then the conditions mentioned above should be considered and the patient re-evaluated (Karsnitz, 2013).

#### 1.9 Prognosis

Delayed initiation of treatment has been associated with a mortality rate of approximately 17%. Death rate is reduced to 2% with early recognition and appropriate treatment. Caesarean deliveries have a 25-fold increase in endometritis-related mortality (Meaney-Delman *et al*., 2015).

### 2. Chronic Endometritis

#### 2.1 *Definition and histopathology*

Chronic endometritis (CE) is a disorder of prolonged, continuous, mild endometrial inflammation, which is characterized by plasma cell infiltration into the endometrial stromal area. The prevalence of chronic endometritis is often underestimated because it is a condition difficult to diagnose. According to the literature, the prevalence of chronic endometritis ranges from 0.2% to 46%, depending on patient profile and biopsy method. Kushnir *et al*. (2016) found that 45% of infertile patients had chronic endometritis, especially those with recurrent implantation failure.

Histopathology is characterised by superficial endometrial lining oedema, abnormally increased stromal cell density, asynchronous maturation between stroma and epithelium, endometrial infiltration with plasma cells (Endometrial stromal plasma cells/ESPCs). Presently, there is no standardized or accepted definition for chronic endometritis, but the presence of numerous endometrial stromal plasma cells (ESPCs) is the most sensitive and specific finding for diagnosing and defining this disease (Kitaya & Yasuo, 2011; Kitaya *et al*., 2018).

#### 2.2 Clinical Presentation

Chronic Endometritis has ill-defined symptoms such as pelvic discomfort, spotting and leucorrhoea. Patient may also complain of hypomenorrhoea, secondary amenorrhoea, and infertility.

#### 2.3 Etiopathogenesis

The uterus consists of three layers: the perimetrium or outer serosal layer of the uterus, the middle smooth muscle called the myometrium, and the innermost layer known as the endometrium. The endometrium has two layers: an inner glandular layer, which is functional with a stroma that is formed by supporting connective tissue and an outer basal layer that provides raw material for regenerating the overlying functional layer after each menstrual cycle.

Under normal conditions, the monthly cycling endometrium is infiltrated by a wide variety of leucocytes (immunocompetent cells), which include NK cells, macrophages, and T cells. The density and composition of these immunocompetent cells vary cyclically across the menstrual cycle. This fluctuation in local leukocyte subtypes is thought to contribute to pathogenesis by affecting tissue remodelling that makes the endometrium receptive in nature.

Correct tissue diagnosis of chronic endometritis has been considered demanding and time-consuming. Recently, focus has shifted to the potential association between chronic endometritis and poor reproductive outcome.

Chronic endometritis is most commonly caused by chronic bacterial infection of the innermost lining of the uterus. In the South-Asian subcontinent, *Mycobacterium tuberculosis* causes chronic granulomatous endometritis, a subtype of chronic endometritis. This is characterized by multiple caseating granulomas and lymphocyte infiltrates including endometrial stromal plasma cells. In developing nations, it is very common to have infertile individuals tested for endometrial aspirate histopathology, AFB (acid fast bacilli) stain, and PCR of *M. tuberculosis* testing to detect genital tuberculosis, a very common cause of infertility, is also performed. Patients are treated with anti-tubercular therapy prior to infertility treatment. Patients with chronic granulomatous endometritis caused by *M. tuberculosis* generally develop Asherman’s syndrome intrauterine adhesions because of endometrial injury, which leads to secondary amenorrhoea and endometrial cause of infertility.

Microorganisms often detected in endometrium with chronic endometritis include *E. coli, Streptococcus, Enterococcus, Staphylococcus, Mycoplasma* spp, *Ureaplasma urealyticum, Gardnerella vaginalis, Proteus, Klebsiella pneumoniae, Pseudomonas aeruginosa, Corynebacterium*, Yeasts (*Saccharomyces* and candida spp), and *Mycobacterium tuberculosis* (Kitaya & Yasuo, 2011).

*C. trachomatis* and *N. gonorrhoeae*, the principle pathogens associated with acute endometritis, are seldom found in chronic endometritis (2% to 8% and 0% to 7%, respectively). These differences in microbiological profiles suggest that acute and chronic endometritis are two distinct entities pathologically.

#### 2.4 Evaluation

On histopathology, chronic endometritis is characterised by endometrial stromal plasma cells, plasma cells with a characteristically high nuclear-cytoplasmic (N/C) ratio, heterochromatin rearrangement in the nucleus classically referred to as the spoke wheel pattern, and basophilic cytoplasm

On routine Hematoxylin and Eosin (H & E) staining, identification of asynchronous stromal and glandular appearance and endometrial eosinophil infiltrates is an easy screening method to look for endometrial stromal plasma cells/ESPCs, but they are not the only histological changes in chronic endometritis (Kitaya & Yasuo 2011; Kitaya *et al*., 2018).

The most reliable diagnostic method for chronic endometritis is immunohistochemistry (IHC) for CD138, which is not just specific but also time saving. CD138 is also called syndecan-1, a transmembrane heparin sulphate proteoglycan and a plasma cell marker.

##### Problems in diagnosing chronic endometritis

Even experienced pathologists have trouble identifying ESPCs on conventional tissue staining. ESPCs are difficult to identify because they closely resemble mononuclear cells (leucocytes) and stromal fibroblasts in the endometrium. Increased stromal cell infiltration into the endometrium interferes with ESPC identification. Technical standards and specifications on CD138 immunostaining for endometrial specimens are currently lacking and there is little international agreement on the subject. Further standardization and improved quality control for CD138 immunostaining is required to make the diagnosis of chronic endometritis more specific. The timing and technique of endometrial aspiration are important for the correct analysis of chronic endometritis. ESPCs may be missed if biopsy samples are inadequate. Literature shows that a diagnosis of chronic endometritis is often possible when tissue samples are taken in the proliferative phase of the endometrium rather than the secretory phase. Therefore, it is necessary to know the phase of the menstrual cycle and the endometrial biopsy volume to accurately diagnose individuals with chronic endometritis. The endometrium of a healthy fertile woman may also show few endometrial stromal plasma cells (ESPCs). It is important to define the minimum amount of the endometrial aspiration sample required and determine the cut-off density of endometrial stromal plasma cells required for histologically identifying chronic endometritis.

##### Microbial Culture

Bacterial culture is one of the most important tools in the diagnosis of chronic endometritis. This technique allows the identification of pathogens and the prescription of targeted therapy.

Limitations of endometrial culture include contamination with vaginal bacteria, limited culturability of fastidious organisms, and delays in culturing bacteria. The use of RT-PCR might alleviate several limitations of conventional culture techniques by providing fast and more accurate profiling of the microorganisms responsible for chronic endometritis (by facilitating the identification of culturable and non-culturable bacterial DNA).

Moreno *et al*. (2018) conducted a prospective cohort study in which endometrial samples were taken from patients suspected for chronic endometritis. A multiplex RT method was used for non-culturable strains and endometrial samples were inoculated onto media containing columbia-colistin-nalidix acid agar with 5% sheep blood, for gram-positive organisms, and MacConkey agar and mannitol salt agar for gram-negative bacteria and Staph aureus. RT-PCR testing was done using specific primers for the nine most common bacteria responsible for causing chronic endometritis. 16S ribosomal RNA sequencing was done and endometrial microbiome profiles were obtained with Next Generation Sequencing. The authors found that RT-PCR testing was useful in the molecular diagnosis of chronic endometritis from endometrial samples, since it uses a comprehensive panel of primers to detect the most commonly involved microorganisms. RT-PCR can detect intrauterine microbiome when histology is negative; when histology is positive, RT-PCR can also inform the choice of target therapy. The limitations of chronic endometritis diagnosis using individual classic techniques and their misleading results were evident in this study, in which only 13 of 65 (20%) of the samples/patients analysed presented concordant results using all three diagnostic methods (Moreno *et al*., 2018).

##### Imaging

HSG may be performed to evaluate tubal status in infertile women. HSG is contraindicated if acute infection is suspected. If HSG is performed during acute infection, acute endometritis manifests with an irregular endometrial cavity and intravasation of contrast into the vascular and lymphatic system (Shah *et al*., 2015). Chronic endometritis presents with calcification of the endometrium on plain film and irregularity of the endometrial cavity on contrast film due to fibrosis, scarring, and calcification. HSG is a useful diagnostic tool to detect intrauterine adhesions or Asherman’s syndrome, which are seen as irregular filling defects (linear, angulated, or stellate shaped) with well-defined borders. If there are grade IV intrauterine adhesions, then the cavity fails to distend and presents with significant reductions of volume and capacity (Shah *et al*., 2015).

Klein *et al*. (1976) described the criteria to diagnose Genital TB based on the following HSG findings: intrauterine adhesions in the absence of history of curettage or surgical termination of pregnancy, multiple constrictions in the fallopian tubes, obstruction of the isthmo-ampullary level of fallopian tubes, and calcification of lymph nodes or irregular linear/nodular calcifications in the adnexal area.

On ultrasound (USG), patients with chronic endometritis may present with a thin endometrium with hyperechoic areas that represent foci of calcification or fibrosis (Shah *et al*., 2015), irregular endometrial lining; 4D USG shows irreversible sequelae of fibrosis and scarring or intrauterine adhesions (Asherman’s Syndrome). On saline infusion sonography, intrauterine adhesions appear as linear echogenic bridges in the fluid filled uterine cavity; this is a good technique to diagnose Asherman’s syndrome. Deformed uterine cavity can give rise to various abnormal shapes and the HSG appearance may mimic a pseudo-unicornuate uterus or a T-shaped uterus (Shah *et al*., 2015).

Patients with suspected genital tuberculosis, secondary amenorrhoea or infertility or with associated tubo-ovarian masses, may undergo imaging like USG, MRI, CT scan or PET- CT scan. A study by Sharma *et al*. (2012) found that the detection rates of tubo-ovarian masses with PET CT was similar to CT or MRI, but the characterization of adnexal masses was less accurate than in CT or MRI. PET-CT was equally precise in detecting the presence or absence, localization, and activity of tubo-ovarian (TO) masses, when compared with laparoscopy or laparotomy.

##### Hysteroscopy

Hysteroscopy can be a useful screening tool for chronic endometritis (Féghali *et al*., 2003; Johnston-MacAnanny *et al*., 2010). Hysteroscopic findings seen in chronic endometritis are endometrial micropolyposis, described as multiple 1-2 mm protrusions or polyps arising from the endometrium with associated endometrial stromal thickening and oedema, found normally in 11% patients on routine hysteroscopy and present in 50 to 67% of women with infertility having either recurrent pregnancy loss (RPL) or repeated implantation failure (RIF) with tissue diagnosis of chronic endometritis. The correlation between micropolyposis of the endometrium and chronic endometritis is still unclear. Strawberry spots are hyperaemic endometrial areas flushed with a white central point on the endometrium visualised during hysteroscopy. They are found in 65% of women with a tissue diagnosis of chronic endometritis. Endometrial micropolyposis and strawberry spots on hysteroscopy have 16-54% sensitivity and 60-94% specificity for diagnosing chronic endometritis (Cicinelli *et al*., 2005; Johnston-MacAnanny *et al*., 2010). The literature suggests that tissue diagnosis with CD138 immunostaining is better than hysteroscopy in diagnosing chronic endometritis.

Cicinelli *et al*. (2019) conducted a study to propose diagnostic criteria for chronic endometritis based on hysteroscopy, and validated the proposed criteria in a RCT. Hysteroscopy is considered as the standard procedure in the evaluation of the uterine cavity in patients with intrauterine conditions such as infertility, RPL, myomas, abnormal uterine bleeding, etc. (Cicinelli *et al*., 2019). Cicinelli *et al*. (2005) were the first to find the combination of stromal oedema, micropolyps, and focal hyperaemia in subjects with chronic endometritis, as later confirmed by other authors.

The authors found that strawberry spots on the endometrium were frequently seen in patients with chronic endometritis, either as an isolated finding or in association with endometrial alterations (e.g. focal hyperaemia). Haemorrhagic spots are caused by chronic inflammation and lead to vascular damage. Chronic inflammation can lead to fibrinoid degeneration or, in rare occasions, to the formation of thrombi in the vessel wall leading to angiopathy/vasculopathy, in patients with chronic endometritis.

On hysteroscopy, chronic granulomatous endometritis or genital tuberculosis present as pale-looking cavity, tubercles, caseous nodules, and/or intrauterine adhesions or Asherman’s syndrome (Sharma *et al*., 2008; 2009). A study by Kumar & Kumar (2007) documented a shining star sign on hysteroscopy in patients with genital tuberculosis, which actually were white caseous nodules as stars shining against the blue background of methylene blue dye. Hysteroscopy in patients with genital tuberculosis is difficult and poses a significant risk of complication. Patients with the condition have a small shrunken cavity, and should preferably undergo laparoscopic guided operative or non-operative hysteroscopy procedure performed by an expert gynaecologist (Sharma *et al*., 2011).

#### 2.5 Treatment / Management

Treatment of chronic endometritis revolves primarily around oral antibiotics, depending on the culture and gram stain findings of the endometrial aspiration/biopsy; endometrial aspiration is repeated after treatment. There is no defined antibiotic regimen for chronic endometritis. Different antibiotics and dosages have been prescribed. Endometrial receptivity tends to improve after antibiotic therapy.

Various antibiotic regimens have been tried. The first line regimen is Tab Doxycycline 100mg BD for 14 days. Second-line therapy includes ciprofloxacin and metronidazole 500 mg OD for two weeks or ofloxacin 400 mg OD for two weeks and metronidazole 500 mg OD for two weeks.

Cicinelli *et al*. (2015) described the prescription of specific antibiotic regimens to infertile chronic endometritis patients according to their microbiologic profiles. Patients with Gram-negative and Gram-positive bacteria were treated with ciprofloxacin 500 mg twice daily for 10 days and amoxicillin-clavulanic acid combination 2 g once a day for 8 days. Patients with Mycoplasma or Ureaplasma infection were administered josamycin 2 g per day for 12 days, while minocycline 200 mg per day for 12 days was given to resistant cases. Chronic Endometritis has been found in 25% of patients even after three courses of oral antibiotic therapy, which indicates reasonable efficacy of oral antibiotic therapy for chronic endometritis. Cicinelli *et al.* (2015) also reported that clinical pregnancy rate and eventually live birth rate in IVF patients was significantly higher in patients showing response to oral antibiotic treatment (65% and 60.8%, respectively) than in patients with persistent chronic endometritis (33% and 13.3%, respectively).

Few authors have described a correlation between antibiotic administration and IVF outcomes in patients with chronic endometritis. Vitagliano *et al*. (2018) conducted a meta-analysis to study the effects of antibiotic treatment for chronic endometritis on the outcome of IVF in patients with recurrent implantation failure. Patients having cured chronic endometritis showed higher clinical pregnancy rates (OR, 4.02), live birth rates (OR, 6.81), and implantation rates (OR, 3.24) than patients with persistent endometrial infection (Vitagliano *et al*., 2018).

Sfakianoudis *et al*. (2018) published a retrospective case series and found three patients with repeated implantation failures (RIF) and unsuccessful treatment of chronic endometritis. Based on antibiogram results, accurate antibiotic treatment was prescribed. Patients with chronic endometritis after oral antibiotic treatment were given an infusion of *intrauterine antibiotic.* Two of the three patients reported on-going, complication-free pregnancies at 19 weeks and 20 weeks. Studies with animal models have described decreased presence of microbes in the uterine cavity and enhanced local immune defence in subjects treated with intrauterine infusion of antibiotics. Treatment resulted in the restoration of the endometrium (Sfakianoudis *et al*., 2018).

##### Treatment of chronic granulomatous endometritis or tubercular endometritis is anti-tubercular therapy (ATT)

All new cases that are drug sensitive and are either diagnosed clinically or confirmed microbiologically should receive combination therapy. Medical therapy with anti-tubercular drugs for 6-9 months is effective for cases of genital tuberculosis (Arora *et al*., 1992). In a randomized controlled trial, six months of ATT was found to be equally effective as nine months therapy for genital tuberculosis (Sharma *et al*., 2016).

*Directly observed treatment short course strategy (DOTS)* is recommended by the WHO and is the preferred mode of treatment. Four drugs - isoniazid (H), rifampicin (R), pyrazinamide (Z) and ethambutol (E) - are given for two months (HRZE), followed by H, R and E (HRE) daily for four months. Daily treatment is given under direct supervision. A 60 kg adult should receive the following dosage of the respective drugs: isoniazid 300mg/day, rifampicin 600mg/day, pyrazinamide 1600mg/day, and ethambutol 1200mg/day. Patients may also take prescription combination kits without direct supervision (non-DOTS treatment) (Sharma *et al*., 2016).

Sharma *et al*. (2016) evaluated the effects of anti-tubercular therapy on the endometrium of females with genital tuberculosis and found that patients resumed having regular menstrual cycles. On histopathology, AFB and epithelioid granulomas disappeared. On USG evaluation, endometrial thickness improved from 7mm to 7.5 mm. On hysteroscopy, post ATT patients had better-looking cavities and less pale-looking cavities. Prevalence of intrauterine adhesions/Asherman’s syndrome was 62% before treatment with ATT and decreased to 28.7% after treatment with ATT. The endometrial cavity improved mainly in patients with grade I adhesions, from 34% to 2.1%, (*p*<0.001). There was no improvement in higher grade intrauterine adhesions/Asherman’s syndrome with ATT. Therefore, early detection of tubercular endometritis is very important. Early ATT improved menstrual cycle, endometrial thickness, and reduced incidence of grade I adhesions, an indication that it may improve the reproductive outcomes of these patients. Individuals with advanced disease do not improve with ATT and have poor reproductive outcomes (Bhagwan Sharma *et al*., 2016). Another study by Sharma and Singh *et al*. looked into the differences in efficacy of 6-month or 9-month ATT in patients with genital tuberculosis and found that there was no difference in complete cure, recurrence, or pregnancy rates between 6-month and 9-month ATT administration (Sharma *et al*., 2016).

#### 2.6 Chronic Endometritis and Reproductive Outcomes

If chronic endometritis is not diagnosed in a timely and systematic manner, it may become one of the factors leading to recurrent IVF failure. Recurrent IVF failure because of chronic endometritis causes psychological stress, frustration, and a financial burden for couples, potentially leading to higher risk complications.

##### Assisted reproduction in females with genital tuberculosis

Patients with genital tuberculosis have a high incidence of infertility despite ATT, with conception rates of only 19.2 per cent (Tripathy & Tripathy, 2002). Patients with blocked tubes but a normal endometrium submitted to IVF-ET enjoy better fertility outcomes (Parikh *et al*., 1997; Jindal *et al*., 2012). A study by Parikh *et al.* (1997) documented a pregnancy rate of 16.6% after IVF-ET and ATT in patients with normal endometria. Jindal *et al.* (2012) observed a population of women with tuberculosis and found a pregnancy rate of 17.3% in IVF-ET as compared to only 4.3% after fertility enhancing surgery for tubal block. Gestational surrogacy is an option for patients with a normal ovarian reserve and intrauterine adhesions (endometrium destroyed with genital tuberculosis). Studies have documented a viable delivery rate of 50 per cent with gestational surrogacy (Sharma *et al*., 2018). Adoption is advised if ovaries are destroyed with diminished ovarian reserve (Neonakis *et al*., 2011; Sharma, 2015).

The reasons of infertility due to chronic endometritis include decreased endometrial receptivity due to direct effect of microbes, presence of lymphocyte subtypes in the endometrium leading to an abnormal micro-environment milieu which hampers endometrial receptivity, local immune response, abnormal milieu in the endometrium for the recruiting circulating B cells, along with increased levels of inflammatory markers in the endometrium. Diseased endometria of patients with chronic endometritis do not respond to ovarian steroid treatment, which is given to patients during IVF cycles to improve endometrial receptivity (Kimura *et al*., 2019). Patients with chronic endometritis due to tuberculosis with advanced intrauterine adhesions/Asherman’s syndrome do not respond to estradiol for endometrial preparation before embryo transfer. Singh *et al*. (2020) conducted a revolutionary study in patients with Asherman’s syndrome (AS) and evaluated the role of BM-derived autologous stem cell therapy in endometrial regeneration and restoration of menstruation and fertility in refractory cases of AS and endometrial atrophy (EA). BM-derived mononuclear stem cells were instilled into the sub-endometrial zone of 25 patients followed by oral oestrogen therapy for 3 months. Menstrual flow and endometrial thickness (ET) were evaluated at intervals of 3, 6, and 9 months and 5 years. Mean ET (mm) before stem cell transfer was 3.3±1.0 mm. At the end of 3 months, there was a significant increase in ET (mm) to 5.1±1.9 (*p*=0.001), but there was no significant change at 6 months, at 9 months, or at the end of 5 years. Menses resumed in 85% of the secondary amenorrhoea patients. Three patients had successful pregnancy outcomes. The authors concluded that intrauterine stem cell treatment is a promising novel approach for refractory cases of Asherman’s syndrome (most common cause in developing nations being tubercular endometritis).

A recent prospective study by Liu *et al*. (2019) reported that patients with intrauterine adhesions caused by chronic endometritis had an imbalance of endometrial fibrosis homeostasis and a higher recurrence rate of adhesions, which lead to poor endometrial receptivity, poor pregnancy rate, and lower live birth rate. Chronic inflammation affects the steady-state balance of endometrial healing and repair and causes fibrosis, which leads to the development and recurrence of intrauterine adhesions. This in totality affects endometrial receptivity and hampers the reproductive outcomes of patients with intrauterine adhesions.

A study by Zanozin *et al*. (2016) reported that fibrosis tissue remodelling is blocked in chronic endometritis (CE), which leads to increased endometrial stromal sclerosis, poor endometrial receptivity, and subsequently infertility. The most important marker of endometrial receptivity is avb3 (Illera *et al*., 2003). Liu *et al*. (2019) found that the average avb3 in the CE group was lower than that in the non-CE group (*p*<0.001), indicating that patients with CE have decreased endometrial receptivity. CE can affect endometrial receptivity in patients with hydrosalpinx (Bao *et al*., 2017).

Chronic endometritis is common in patients with unexplained infertility. The diagnosis and treatment of chronic endometritis in patients with unexplained infertility may improve pregnancy rates (Cicinelli *et al*., 2018). Liu *et al*. (2019) reported that pregnancy and live birth rates were lower in the chronic endometritis group than in the non-CE group, a finding that implies that CE hampers the reproductive prognosis of patients with intrauterine adhesions.

Animal studies have shown that endometrial bacterial colonization is directly associated with disruption of endometrial integrity, influx of neutrophils and secretion of inflammatory cytokines, and chronic inflammatory response. These lead to endocrine dysfunction of the endometrium, thereby disrupting the embryo-endometrium interaction and hampering the implantation process.

## CONCLUSION

Early identification of acute or chronic (like tubercular) endometritis and antibiotic/anti-tubercular treatment are very important, since they can have long term implications on the reproductive outcome of patients with these conditions. The aetiology of chronic endometritis is mainly microbiological, but its origin still needs to be elucidated. Chronic endometritis causes impaired decidualization as well as local immune abnormalities in the endometrium, which result in implantation failure. For patients with advanced intrauterine adhesions, intrauterine stem cell treatment is also a promising novel approach for improving their fertility outcomes. Larger prospective and randomised trials are required for validating this novel technique.
